# Use of antenatal corticosteroids for preterm birth in Latin America: providers knowledge, attitudes and practices

**DOI:** 10.1186/1742-4755-10-4

**Published:** 2013-01-29

**Authors:** Alicia Aleman, Maria L Cafferata, Luz Gibbons, Fernando Althabe, Jose Ortiz, Xochitl Sandoval, Nicolás Padilla-Raygoza, José M Belizán

**Affiliations:** 1 Department of Preventive Medicine, School of Medicine, University of Uruguay, Montevideo, Uruguay; 2 Clinical and Epidemiological Research Unit Montevideo, Montevideo, Uruguay; 3 Institute for Clinical Effectiveness and Health Policy, Buenos Aires, Argentina; 4 School of Medical Sciences, University of Cuenca, Cuenca, Ecuador; 5 School of Medicine, Evangelic University of El Salvador, San Salvador, El Salvador; 6 Departamento de Enfermería y Obstetricia, División de Ciencias de la Salud e Ingenierías, Campus Celaya Salvatierra, Universidad de Guanajuato, Celaya, Mexico

**Keywords:** Antenatal corticosteroids, Preterm birth, Respiratory distress syndrome/prevention

## Abstract

**Background:**

Antenatal corticosteroids administered to women at risk of preterm birth is an intervention which has been proved to reduce the risk of respiratory distress syndrome, intraventricular hemorrhage, and neonatal mortality. There is a significant gap in the literature regarding the prevalence of the use of antenatal corticosteroids in Latin American countries and the attitudes and opinions of providers regarding this practice. The aim of this study was to assess the knowledge, attitudes and practices of health care providers regarding the use of antenatal corticosteroids in women at risk of preterm birth in Latin America.

**Methods:**

This was a multicenter, prospective, descriptive study conducted in maternity hospitals in Ecuador, El Salvador, Mexico and Uruguay. Physicians and midwives who provide prenatal care or intrapartum care for women delivering in the selected hospitals were approached using a self-administered questionnaire. Descriptive statistics was used.

**Results:**

The percentage of use of ACT in threatened preterm labour (TPL) reported by providers varies from 70% in Mexico to 97% in Ecuador. However, 60% to 20% of the providers mentioned that they would not use this medication in women at risk and would limit its use when there was a threatened preterm labour. In only one country recommended regimens of antenatal corticosteroids are followed by around 90% of providers whereas in the other three countries recommended regimens are followed by only 21%, 61%, 69% of providers. Around 40% of providers mentioned that they would administer a new dose of corticosteroids again, regardless the patient already receiving an entire regimen. Between 11% and 35% of providers, according to the countries, mentioned that they do not have adequate information on the correct use of this medication.

**Conclusions:**

This study shows that the use of this intervention could be improved by increasing the knowledge of Latin American providers on its indications, benefits, and regimens.

## Background

Preterm birth is the main cause of four million neonatal deaths each year worldwide, 99% of which occur in low-and middle-income countries [1].

Antenatal corticosteroid administered to women at risk of preterm birth is an intervention which has proved to reduce the risk of respiratory distress syndrome, intraventricular hemorrhage, and neonatal mortality by 50% or more [2-4]. There is a significant gap in the literature regarding the prevalence of the use of antenatal corticosteroids in Latin American countries; very few studies have been published and the results vary widely from 4% to 37% [5-8]. In addition, there is an urgent need to identify the cultural and geographical factors that contribute to the lack of use in order to develop specific interventions to increase the appropriate use of antenatal steroids.

We recently published an article showing a varied pattern of the use of prenatal corticosteroids among women delivering prematurely in hospitals in 3 Latin American countries. Ecuador had the lowest use (34.8%), followed by El Salvador (54.6%), and Uruguay (71%) and the maternal characteristics associated with their use [9].

The aim of this study was to assess the knowledge, attitudes, practices and barriers of the health care providers regarding the use of antenatal corticosteroids in women at risk of preterm birth in Ecuador, El Salvador, Mexico and Uruguay.

## Methods

### Study design and population

This was a multicenter, prospective, descriptive study targeted at health providers who provided antenatal or intrapartum care to women delivering in hospitals in Ecuador, El Salvador, Mexico and Uruguay.

The study was conducted in four public hospitals in Ecuador (in the cities of Guayaquil, Cuenca, Loja and Quito), three public and one social security hospital in El Salvador (in the cities of San Salvador, 2 hospitals, San Miguel and Santa Ana), one public and one social security hospital in Mexico (in Guanajuato State), and five public hospitals in Uruguay (in the cities of Montevideo, Paysandú, Salto and Tacuarembó). These countries were selected as geographical examples of four sub-areas of the region (Andean area, Central America, North America and the South Cone, respectively.

Health-care providers who worked in the participating facilities and had the provision of prenatal or intrapartum care as their primary responsibility were included. This included physicians, midwives, nurse practitioners and in some cases medical students. A convenient sample of 5 health care workers per site who provided prenatal care or attended deliveries in the community and referred patients to the participating hospitals were also included.

### Procedures

Hospital providers, including faculty, residents, physicians, midwives and nurses were identified by the hospital director and chiefs of the obstetrics and neonatal divisions as well as by the local coordinator. Community health care providers were identified through patients and other providers’ referral. All eligible providers were invited to participate by signing a consent form.

Scientific meetings, weekly rounds and “on call” sign overs were used to approach the hospital providers. Those who could not be reached by these means were contacted personally.

All eligible providers were given an envelope containing an informative letter about the study, contact information for obtaining further information, the consent form and a self administered questionnaire. All completed forms were returned in a sealed envelope.

Community providers were initially contacted by phone or in person, either by the data collectors or the local coordinator. The survey was delivered in person or, if the provider accepted, by phone. Based on previous studies from Latin American countries and the commitment of the participating institutions, we anticipated a response rate of at least 80% [10].

A self-administered questionnaire was developed by the research team for this study. It was sent in a preliminary version to a panel of experts (obstetrics, midwives, neonatologists, family doctors) from the participating hospitals. Based upon the panel's critique, the research team built the final instrument. The questionnaire was piloted in a sample of providers from the participating hospitals in each country in order to reach cultural adequacy.

The questionnaire included 45 items including questions related to socio-demographic and professional characteristics, clinical practice characteristics, knowledge on the magnitude of the problem of preterm birth, prevalence of the use of antenatal corticosteroids, attitudes towards the use of antenatal corticosteroids, current practices with respect to antenatal corticosteroids, knowledge on side effects and contraindications, knowledge on types of corticosteroids, doses and frequency, the moment of pregnancy when it is indicated, common barriers to the appropriate use of antenatal steroids and issues of providers’ opinions on the magnitude and importance of the topic. Some clinical situations were also posed to the providers to assess their behaviours under these circumstances.

### Data management

The country coordinator from each country periodically collected all forms from the participating hospitals. Each form was photographed by the data management coordinator with a digital camera provided by the study coordination, and then transmitted to special email accounts at the Central Data Management Unit using the SSL (Secure Sockets Layer) protocol, with a hosting provider digital certificate. Access to the system was limited to authorized individuals in each participating country and in the Central Data Management Unit located at the Clinical and Epidemiological Research Unit at Montevideo, Uruguay (UNICEM), using a user name and password. The data was entered in a secure data management system (DMS), specifically designed for this study and fully compliant with good clinical practices. A double data entry was performed on a 15% random sample of the data forms to assess quality of data entry. To preserve the confidentiality of the participants, personal identifiers were not included in the data forms or transmitted to the data center. Hospital data collectors signed a confidentially agreement at the beginning of the study.

### Data analysis

Sample size was calculated assuming a frequency of around 80% on the reply to the major outcome: administration of antenatal corticosteroids (ACT) on women with threatened preterm labour (TPL). Assuming a frequency of around 75% and a sample of 80 providers in each country the true value will fluctuate between 65% and 85%.

The total response rate was calculated and data on refusal rate (active and passive) and other factors associated with recruitment efforts were calculated.

A descriptive analysis of providers’ socio-demographic and professional characteristics was performed for each country as well as globally. A binomial 95% confidence interval of the proportions of providers who start prescribing antenatal corticosteroids before 24 weeks, between 24 to 27 weeks and between 28 to 34 weeks for each country was reported. In order to make the comparison of the proportions of providers that used antenatal corticosteroids in different obstetric situations even when women did not have a threatened preterm labour but they were at risk of delivering preterm, Marascuilo procedure was used. This procedure enables to simultaneously test the differences of all pairs of proportions.

This study was approved by the Institutional Review Board in each country. (IORG0002991 Facultad de Medicina, Universidad de la Republica del Uruguay, IRB00002438 - Universidad Central del Ecuador, Facultad de Ciencias Medicas del El Salvador, IRB00004952 - Hospital Nacional Rosales de Ecuador and Bioethical Committee, Facultad de Enfermería y Obstetricia de Celaya de la Universidad de Guanajuato, Mexico).

## Results and discussion

A total of 353 health providers answered the questionnaire. Older ages were seen in Ecuadorian providers. In all the countries the majority of them were medical doctors. Uruguay shows the highest proportion of midwives (21.5%). Years assisting deliveries varied between 5 and 14 among the countries and the mean number of deliveries assisted by month by the professional varies from 4.5 in Mexico to 20 in El Salvador (Table 1). The response rate was 98% (59/60) in Mexico (Celaya), 96% (109/113) in El Salvador, 65% (121/186) in Uruguay and 48% (64/133) in Ecuador.

**Table 1:** Socio-demographic characteristics of respondents

		**El Salvador (N=109)**		**Mexico (N=59)**		**Ecuador (N=64)**		**Uruguay (N=121)**	
		**n**	**(%)**	**n**	**(%)**	**n**	**(%)**	**n**	**(%)**
**Age **(mean and S.D.)		33.8	(8.3)	42.0	(11.7)	44.3	(13.3)	38.1	(8.4)
**Gender **(Male)		50	(45.9)	36	(61.0)	36	(56.3)	50	(41.3)
**Profession**	Medical Doctor	103	(94.5)	53	(89.8)	60	(93.8)	95	(78.5)
	Medicine Student	6	(5.5)	0	(0.0)	0	(0.0)	0.0	(0.0)
	Midwives	0	(0.0)	6	(10.2)	1	(1.6)	26	(21.5)
	Nurse	0	(0.0)	0	(0.0)	3	(4.7)	0.0	(0.0)
**University affiliation**		18	(16.5)	10	(16.9)	25	(39.1)	40	(33.1)
**Years assisting deliveries **(median and IQR)		5	(3-8)	14	(5.3 – 23.8)	13	(4-26)	9	(3-14)
**Deliveries assisted by month **(median and IQR)		20	(10-30)	4.5	(2 -10)	8	(4-25)	12	(7.5-20)

IQR: Inter Quartile range.

The majority of providers started providing ACT beyond 28 weeks of amenorrhea with the exception of Uruguay. (Table 2).Most Uruguayan professionals (80. 2%) reported the prescription of ACT between 24 to 27 weeks of gestational age.

**Table 2:** Use of Corticosteroids reported by the providers

		**El Salvador (N=109)**		**Mexico (N=59)**		**Ecuador (N=64)**		**Uruguay (N=121)**	
		**n/N (%)**	**[95% CI]**	**n/N (%)**	**[95% CI]**	**n/N (%)**	**[95% CI]**	**n/N (%)**	**[95% CI]**
Start prescribing antenatal corticosteroids	Before 24 weeks	1/102 (1.0)	[0.0 – 5.3]	4/41 (9.8)	[2.7 – 23.1]	0/60 (0.0)	[0.0 – 6.0]	1/101 (1.0)	[0.0 – 5.4]
	24 to 27	52/102 (51.0)	[40.9 – 61.0]	8/41 (19.5)	[8.8 – 34.9]	21/60 (35.0)	[23.1 – 48.4]	81/101 (80.2)	[71.1 – 87.5]
	28 to 34	49/102 (48.0)	[38.0 – 58.2]	29/41 (70.7)	[54.5 – 83.9]	39/60 (65.0)	[51.6 – 76.9]	19/101 (18.8)	[11.7 – 27.8]

The percentage of providers that reported prescribing antenatal corticosteroids between 24 to 27 weeks was also statistically different between El Salvador and Mexico (51% vs 19.5% p<0, 05).

In El Salvador, Mexico and Ecuador the most ACT used was Betametasone, while in Uruguay Dexametasone was exclusively used. Two doses of 12 mg of Betametasone were used by 90% of providers in El Salvador and 60% and 21% in Ecuador and Mexico, respectively. 61% of the Uruguayan providers used 4 doses of 6 mg of dexametasone (data not shown).

The percentage of providers that answered that they administered antenatal corticosteroids on women with threatened preterm labor (TPL) varied from 70% in Mexico to 97% in Ecuador. Situations that diminished the frequency of administration on women with TPL varied between countries. In Mexico a marked reduction of corticosteroids provision by providers was mentioned when women presented complications such as diabetes, hypertension and any infection. A similar but less marked pattern was shown in Ecuador. In El Salvador and Uruguay minor changes in the number of providers limiting administration due to a diversity of events was seen (Table 3).

**Table 3:** Frequency of providers that use antenatal corticosteroids in threaten preterm labor

	**El Salvador (N=109)**		**Mexico (N=59)**		**Ecuador (N=64)**		**Uruguay (N=121)**		Differences between Countries*
	**n/N**	**(%)**	**n/N**	**(%)**	**n/N**	**(%)**	**n/N**	**(%)**	
Women with threatened preterm labor (TPL)	100/106	(94.3)	35/50	(70.0)	58/60	(96.7)	106/121	(87.6)	- Mexico differs from El Salvador and Ecuador
Women with TPL and									
Rupture of membranes	100/105	(95.2)	28/45	(62.2)	49/59	(83.1)	105/121	(86.8)	-Mexico differs from El Salvador and Uruguay
Diabetes	90/101	(89.1)	21/45	(46.7)	36/56	(64.3)	97/121	(80.2)	-Mexico differs from El Salvador and Ecuador - El Salvador differs from Ecuador
Hypertension	97/102	(95.1)	23/43	(53.5)	40/55	(72.7)	100/121	(82.6)	- El Salvador differs from the rest Mexico differs from Uruguay
Multiple pregnancy	104/107	(97.2)	38/49	(77.6)	58/60	(96.7)	106/121	(87.6)	- El Salvador differs from Mexico and Uruguay
									- Mexico differs from Ecuador
Upper urinary infection	94/104	(90.4)	18/41	(43.9)	38/56	(67.9)	99/121	(81.8)	- El Salvador differs from Mexico and Ecuador
									- Mexico differs from Uruguay
Lower urinary infection	88/103	(85.4)	17/41	(41.5)	43/56	(76.8)	100/121	(82.6)	- Mexico differs from the rest
Asymptomatic bacteriuria	86/102	(84.3)	16/41	(39.0)	46/57	(80.7)	99/121	(81.8)	- Mexico differs from the rest
Chorioamnionitis	90/105	(85.7)	18/41	(43.9)	28/57	(49.1)	95/121	(78.5)	- El Salvador and Uruguay differ from Mexico and Ecuador

*The Marascuilo test was used to test the differences between countries.

Providers were asked about their behaviour in situations leading to the provision of ACT in the absence of TPL (Table 4). In general with small variations the providers in the countries followed similar behaviours. ACTs are mentioned to be frequently provided in multiple pregnancies, in preterm premature rupture of membranes, and in haemorrhage in the second half of pregnancy. Previous preterm birth and preeclampsia without TPL was mentioned as an indication of ACT by more than 50% of the professionals in all the countries. Other indications leading to ACT administration but with less frequency are: intrauterine growth restriction, hypertension, diabetes, and urinary infection (Table 4).

**Table 4:** Providers prescribing antenatal corticosteroids in several obstetric situations different from a threatened preterm labor

	**El Salvador (N=109)**		**Mexico (N=59)**		**Ecuador (N=64)**		**Uruguay (N=121)**		Differences between Countries*
	**n/N**	**(%)**	**n/N**	**(%)**	**n/N**	**(%)**	**n/N**	**(%)**	
Multiple pregnancy	92/106	(86.8)	33/44	(75.0)	51/59	(86.4)	101/106	(95.3)	- Mexico differs from Uruguay
Preterm premature rupture of membranes	98/106	(92.5)	30/43	(69.8)	46/56	(82.1)	100/106	(94.3)	- Mexico differs from El Salvador and Uruguay
Haemorrhage in the second half of pregnancy	91/106	(85.8)	23/37	(62.2)	38/49	(77.6)	86/106	(81.1)	- No difference between countries
Pre-eclampsia	82/104	(78.8)	22/42	(52.4)	30/47	(63.8)	72/106	(67.9)	- El Salvador differs from Mexico
Intrauterine growth restriction	49/101	(48.5)	17/40	(42.5)	29/48	(60.4)	70/106	(66.0)	- No difference between countries
Previous preterm birth	70/105	(66.7)	24/38	(63.2)	36/52	(69.2)	63/106	(59.4)	- No difference between countries
Upper urinary infection	46/101	(45.5)	16/39	(41.0)	22/45	(48.9)	55/106	(51.9)	- No difference between countries
Hypertension	47/100	(47.0)	16/39	(41.0)	24/44	(54.5)	36/106	(34.0)	- No difference between countries
Diabetes	36/98	(36.7)	10/39	(25.6)	19/45	(42.2)	30/106	(28.3)	- No difference between countries
Lower urinary infection	32/99	(32.3)	16/38	(42.1)	21/44	(47.7)	25/106	(23.6)	- Ecuador differs from Uruguay
Asymptomatic bacteriurua	29/99	(29.3)	8/36	(22.2)	22/43	(51.2)	22/106	(20.8)	- Ecuador differs from Mexico and Uruguay

*The Marascuilo test was used to test the differences between countries.

Providers were faced with different clinical situations to assess their behavior. More than a quarter of them in El Salvador and Ecuador and more than half of them in Mexico replied that they would not give ACT to a healthy woman with a gestational age of 27 weeks, hospitalized due to TPL, meanwhile almost all of the Uruguayan providers would give ACT in the same situation (Table 5). Around two thirds of providers in El Salvador, Ecuador and Uruguay would not give additional ACT to the woman in this clinical situation after discharge. However, in the case of this woman being hospitalized again, around 40% of providers in these three countries would give an additional ACT dose.

**Table 5:** Providers´ replies to theoretical clinical situations

		**El Salvador (N=109)**		**Mexico (N=59)**		**Ecuador (N=64)**		**Uruguay (N=121)**	
		**n/N**	**(%)**	**n/N**	**(%)**	**n/N**	**(%)**	**n/N**	**(%)**
**CLI NICAL SITUATION 1**									
Prescribe antenatal corticosteroids to a healthy women in a gestational age of 27 weeks who is hospitalized due to TPL		81/108	(75.0)	24/51	(47.1)	45/61	(73.8)	102/105	(97.1)
The woman is discharged after 72 hs. The TPL is arrested. ¿Which is the indication?	A doses every month	5/105	(4.8)	5/41	(12.2)	0/56	(0.0)	3/105	(2.9)
	A doses every two weeks	1/105	(1.0)	1/41	(2.4)	2/56	(3.6)	1/105	(1.0)
	A doses every week	24/105	(22.9)	10/41	(24.4)	8/56	(14.3)	8/105	(7.6)
	No doses	68/105	(64.8)	0/41	(0.0)	40/56	(71.4)	71/105	(67.6)
	Other	7/105	(6.7)	25/41	(61.0)	6/56	(10.7)	22/105	(21.0)
Prescribe antenatal corticosteroids for the same patient if she is hospitalized again due to TPL at the gestational age of 32 weeks		42/107	(39.3)			25/60	(41.7)	50/106	(47.2)
**CLI NICAL SITUATION 2**									
Prescribe antenatal corticosteroids to a healthy women with a gestational age of 25 weeks who is hospitalized due to a rupture of membranes of 4 hours of evolution, without cervical dilatation and signs of infection		48/108	(44.4)	21/48	(43.8)	27/61	(44.3)	82/105	(78.1)
**CLINICAL SITUATION 3**									
Prescribe antenatal corticosteroids to a healthy woman with two previous preterm births at 30 and 32 weeks and with the actual pregnancy with no complications		82/108	(75.9)	25/47	(53.2)	27/61	(44.3)	71/107	(66.4)

Between 20% and 57% of the providers according to the countries replied that they considered there to be barriers in the administration of ACT. One of them is availability of ACT with variations among countries from 7.5% to 32% (Table 6). From 9% to 33% according to the countries mentioned that one barrier is the fear or doubt of the side effects of ACT and similar figures were obtained in relation to misinformation about the correct use of ACT. They already mentioned that some barriers are due to the users such as economic barriers and women´s misinformation about the correct use of ACT.

**Table 6:** Barriers for the use of corticosteroids

	**El Salvador (N=109)**		**Mexico (N=59)**		**Ecuador (N=64)**		**Uruguay (N=121)**	
	**n/N**	**(%)**	**n/N**	**(%)**	**n/N**	**(%)**	**n/N**	**(%)**
**Exists barriers**	60/106	56.6	10/49	20.4	16/60	26.7	34/107	31.8
**HEALTH SYSTEM**								
Corticosteroids availability at hospitals	33/103	32.0	4/47	10.6	7/57	12.3	8/107	7.5
Corticosteroids availability at primary health care centers	57/104	54.8	6/47	12.8	8/56	14.3	27/107	25.5
Availability at night hours	22/100	20.2	7/46	15.2	6/56	10.7	21/107	19.6
**PROVIDERS**								
Fear or doubt about the side effect of the use of corticosteroids	33/100	33.0	7/46	15.2	10/56	17.9	10/107	9.3
Misinformation about the correct use of the corticosteroids	36/103	35.0	9/48	18.8	8/54	14.8	12/107	11.2
**WOMEN**								
Economic barriers to get corticosteroids	52/104	50.0	7/46	15.2	9/57	15.8	17/107	15.9
Lack of personnel who can apply the corticosteroids	24/101	23.8	3/45	6.7	3/55	5.5	19/107	17.8
Fear to use corticosteroids	20/102	19.6	6/46	13.0	8/55	14.5	4/107	3.7
Misinformation about the correct use of the corticosteroids	39/101	38.6	5/45	11.1	9/54	16.7	8/107	7.5

This study shows that in four Latin American countries a vast majority of providers replied that they use ACT in women in TPL varying from 70% in Mexico to 97% in Ecuador. However, in some situations with no contraindications of the use of ACT providers, fluctuating from 20% to 60%, providers said that they will limit the use of ACT on women with TPL. In only one country recommended regimens of ACT is followed by around 90% of providers whereas in the other three countries recommended regimen is followed only by 21, 61, and 69% of providers. Around 40% of providers mentioned that they would administer a new dose of ACT again, regardless of the patient already receiving an entire regimen. Between 11% and 35% of providers according to the countries mentioned that they do not have good information on the correct use of ACT.

The reason as to only dexamethasone being used in Uruguay is because bethametasone is not available to be used for the prevention of preterm birth newborn complications in this country.

Even though the participating countries were selected to represent 4 main Latin American sub regions, and the participating hospitals are reference institutions in their countries, because they were not randomly selected we cannot infer that the observed pattern of use is representative of other hospitals in those countries. However, because we selected influential reference hospitals in the countries’ capital cities, we believe that this might increase the likelihood that the use of corticosteroids in other hospitals may be similar.

Regardless of around 90% of providers reporting that they use ACT in TPL, information obtained from women having preterm births in the same hospitals showed figures of 34.8%, 54.6%, and 71% in Ecuador, El Salvador and Uruguay, respectively of ACT use [9]. Reasons explaining such discrepancy needs to be subject of analysis and actions to improve the reception of ACT on pregnancies that finally end in preterm birth.

Providers´ lack of knowledge on the use of ACT appears in this survey. Many providers limited the use of ACT under certain obstetrics conditions without any support from the literature justifying such behavior. There is also a disparity in the doses and frequencies of ACT. While in one country the vast majority of providers mentioned the prescription of the doses recommended in the literature and in the international and national guidelines, in other countries between 40% and 80% of providers did not mention this correct regimen. There is also no evidence that repeated doses of ACT are of benefit, but 60% of providers faced with a clinical situation said that they would repeat the administration after an episode of ATL [11].

There are many barriers considered by the providers that could be solved by better availability of the drug and also by a better knowledge of providers and users on the benefits of ACT.

As previously stated, ACT is one of the most beneficial interventions in reducing neonatal complications due to preterm birth including neonatal death. In spite of this, its use in low-and-middle income countries is lower than expected [5-9].

Although there were national and institutional guidelines in the participating countries at the time of the survey, they were in some cases not updates and not clear. They did not include a clear and direct recommendation of use what could have influence the underuse and misuse of antenatal corticosteroids.

## Conclusions

This study shows that the use of antenatal corticosteroids should be improved in participating countries. Increasing availability of this medication at primary health care centers seems to be a mayor strategy. On the other hand, increasing the knowledge of Latin American providers on the indications, benefits, and regimens of ATC and improving characteristics of national and institutional guidelines are also needed interventions. The development of clear and evidence-based recommendations and interventions to promote providers adoption of them represent valuables strategies to be implemented. Studies developed in other developing countries have reached the same conclusions [12,13].

### Details of ethics approval

This study was approved by the Institutional Review Board in each country.

Facultad de Medicina, Universidad de la Republica del Uruguay, (IRB0002991) date: 2004.

Universidad Central del Ecuador (IRB00002438), date: 4th April, 2005.

Facultad de Ciencias Medicas del El Salvador (IRB00004952) - .date : 26th September 2006.

Comité de Bioética de la Facultad de Enfermería y Obstetricia de Celaya de la Universidad de Guanajuato, Mexico.

## Competing interests

All the authors declare that they do not have any conflict of interest.

## Authors’ contribution

AA, FA and JMB has the original idea about this study. AA designed the first protocol. AA wrote the final protocol in collaboration with JMB and FA. MLC and AA prepared manuals and forms in collaboration with FA. AA co-ordinated the overall execution of the study. AA , with the support of MLC, JO, XS and NP co-ordinated the implementation of the study at country level. LG wrote the plan of analysis in collaboration with FA and JMB, and did the statistical analysis. All authors participated in the writing of the manuscript. All authors read and approved the final manuscript.

## References

[B1] MacDorman MF Martin JA Mathews MS Hoyert DL Explaining the 2001–02 Infant Mortality Increase: Data from the linked birth/infant data set. National Vital Statistics Reports 53 Available at: http://www.cdc.gov/nchs/data/nvsr/nvsr53/nvsr53_12.pdf, accessed January 12, 2006 15712582

[B2] Royal College of Obstetricians and Gynaecologists (RCOG) Antenatal corticosteroids to reduce neonatal morbidity and mortality 2010 London (UK): Royal College of Obstetricians and Gynaecologists (RCOG)

[B3] Crowley P Prophylactic corticosteroids for preterm birth (Cochrane Review). The Cochrane Library, Issue 3 2002 Oxford: Update Software 10.1002/14651858.CD000065 10796110

[B4] NIH Consensus Development Panel Effect of corticosteroids for fetal maturation on perinatal outcomes JAMA 1995 273 413 418 10.1001/jama.1995.03520290065031 7823388

[B5] Forteza C Díaz Rossello JL Matijasevich A Barros F Morbidity and mortality of very low birth weight (VLBW) infants in Montevideo, Uruguay.In: Abstracts from the XXXIX Annual Meeting of the Latin American Society for Pediatric Research. Colonia del Sacramento, Uruguay, nov. 2001 Pediatr Res 2002 9 467

[B6] Krauss Silva L Pinheiro T Franklin R Oliveira N Assessment of quality of obstetric care and corticoid use in preterm labor Cadernos de Salude Publica 1999 15 4 1 23 10.1590/s0102-311x1999000400016 10633204

[B7] Vallejo Valdivieso N Chinga Sampedro J Sánchez Macías M Tumbaco García R Epidemiología del parto pretérmino y su repercusión en la morbi-mortalidad neonatal registrados en el hospital Dr. Verdi Cevallos Balda / Epidemiología of the childbirth preterm and its repercussion in neonatal morbi-mortality registered in hospital Dr Verdi Cevallos Medicina (Guayaquil) 2002 8 1 36 41

[B8] Colomar M Belizán M Cafferata ML Labandera A Tomasso G Althabe F Belizán JB Prácticas en la atención materna y perinatal realizadas en los hospitales públicos de Uruguay Ginecol Osbstet Mex 2004 72 55 65 15587821

[B9] Riganti AA Cafferata ML Althabe F Gibbons L Segarra JO Sandoval X Belizán JM Use of prenatal corticosteroids for preterm birth in three Latin American countries Int J Gynaecol Obstet 2010 108 52 57 10.1016/j.ijgo.2009.08.022 19892349 PMC3401047

[B10] Vargas Origuel A Leon Ramirez D Zamora-Orozco J Corticoesterides antenatales: Empleo y actitud del personal medico ginecoobstetra Ginecol Obstet Mex 2000 68 29195 11006643

[B11] Crowther Caroline A McKinlay Christopher JD Middleton P Harding Jane E Repeat doses of prenatal corticosteroids for women at risk of preterm birth for improving neonatal health outcomes. Cochrane Database of Systematic Reviews The Cochrane Library, Issue 08, Art. No. CD003935 10.1002/14651858.CD003935.pub3 PMC4170912 21678343

[B12] Pattanittum P Ewens MR Laopaiboon M Lumbiganon P McDonald SJ Crowther CA SEA-ORCHID Study Group Use of antenatal corticosteroids prior to preterm birth in four.South East Asian countries within the SEA-ORCHID project BMC Pregnancy Childbirth 2008 8 47 10.1186/1471-2393-8-47 18925968 PMC2596081

[B13] Tita A Selwyn B Waller K Kapadia A Dongmo S Evidence-based reproductive health care in Cameroon: population-based study of awareness, use and barriers Bull World Health Organ 2005 83 12 895 903 16462981 PMC2626486

